# Management Strategies for Hypertensive Crisis: A Systematic Review

**DOI:** 10.7759/cureus.66694

**Published:** 2024-08-12

**Authors:** Naveed N Khan, Elaf J Zurayyir, Afyaa M Alghamdi, Sara F Alghamdi, Mohammed A Alqahtani, Esra M Abdalla, Najwa S Jurays, Abdullah M Alassiri, Hatoon A Alzahrani, Abdullah A Althabet

**Affiliations:** 1 Internal Medicine, King Salman Armed Forces Hospital, Tabuk, SAU; 2 College of Medicine, Jazan University, Jazan, SAU; 3 Medicine and Surgery, Al Baha University, Al Baha, SAU; 4 College of Medicine, Al Baha University, Al Baha, SAU; 5 College of Medicine, King Khalid University, Abha, SAU; 6 General Practice, University of Khartoum, Khartoum, SDN; 7 Internal Medicine, King Khalid University, Abha, SAU; 8 Medicine, Najran University, Najran, SAU; 9 Medicine, Al Baha University, Al Baha, SAU

**Keywords:** hypertension–malignant, emergency treatment, blood pressure, arterial pressure, acute disease, emergency hypertension, hypertensive crisis

## Abstract

A hypertensive crisis is defined as a sudden and significant rise in blood pressure. The blood pressure reading is 180/120 mmHg or higher. A hypertensive crisis is a medical emergency. It can lead to a heart attack, stroke, or other life-threatening medical problems. Investigating the management of the hypertensive crisis was the goal of this study. English-language articles were collected from 2010 to 2024 demonstrating the management of the hypertensive crisis. Overall, there were 15 articles. Surveys and analyses of national databases were the most widely used methods (n=15). The scientific studies documented (1) all investigative studies or reports that included a hypertensive crisis diagnosis, (2) data integrity and reproducibility, and (3) management studies. Other studies show that acute severe hypertension in the hospital is associated with high rates of mortality and morbidity, particularly with new or worsening end-organ damage. The problem is linked to poor medical adherence, but alarmingly low follow-up rates are likely to contribute to a high recurrence rate. The treatment of acute severe hypertension varies according to the hospital unit (medical ward or intensive care unit), medication, and blood pressure targets or thresholds. Because of a lack of evidence-based guidance, arbitrary blood pressure control targets are used, or blood pressure targets are crudely extrapolated from guidelines intended primarily for outpatient management. Patients with acute aortic dissection need to be administered intravenous esmolol within 5 to 10 minutes in order to lower their blood pressure right away. The goal is to maintain a systolic reading of less than 120 mm Hg. Vasodilators such as nitroglycerin or nitroprusside may be administered if the blood pressure persists following beta blocking. Intravenous administration of clevidipine, nicardipine, or phentolamine is required; the initial dose is 5 mg, with subsequent doses given every 10 minutes as necessary to achieve the desired reduction in blood pressure.

## Introduction and background

Hypertensive emergencies are characterized by sudden and significant increases in blood pressure (BP) that result in damage to organs. This condition may manifest through various symptoms, including pulmonary edema, heart ischemia, neurological impairments, kidney failure, aortic dissection, and eclampsia. Often, these emergencies stem from a failure to adhere to prescribed antihypertensive treatments or from the consumption of sympathomimetic drugs, leading to a rapid escalation in BP beyond the autoregulatory capacity of the body. It's important to note that the specific BP thresholds defining a hypertensive emergency can vary and are somewhat subjective, lacking a universal consensus [1,2].

In the United States, approximately 30% of adults suffer from hypertension (HTN), and among these, 1% to 2% may encounter a hypertensive crisis, encompassing both hypertensive emergencies and urgencies. To accurately assess if an individual is experiencing a hypertensive emergency, a comprehensive history and physical examination are essential. Symptoms warranting additional investigation include headaches, dizziness, changes in mental status, shortness of breath, chest pain, reduced urine production, nausea, or alterations in vision. Identifying the underlying cause of the abrupt increase in BP is crucial to guide the appropriate management strategy [3,4].

Hypertensive crisis refers to an immediate and significant rise in BP over 180/120 mmHg, while hypertensive emergency refers to acute hypertensive target organ damage like myocardial infarction, stroke, or heart failure. These patients must have their BP lowered in an intensive care unit by 25% within one to two hours to prevent further damage. Hypertensive urgencies, on the other hand, are sudden and severe rises in BP without accompanying symptoms of target organ damage. Lowering BP within 24 to 48 hours is necessary to prevent HTN [5].

Hypertensive emergencies were common (6/1,000), with 71.7% presenting with HTN urgency, 19.1% with hypertensive emergency, and 9.2% with hypertensive pseudocrisis. The multinominal logistic regression compared hypertensive emergency to pseudocrisis and urgency conditions. The risk of hypertensive pseudocrisis was thus elevated in patients who reported experiencing any kind of discomfort, other than headaches and chest pain (odds ratio (OR): 55.58; 95% confidence interval (CI): 10.55-292.74), as well as emotional issues (OR: 17.13; 95% CI: 2.80-104.87). Preventing hypertensive pseudocrisis was possible with both a high age (OR: 0.32; 95% CI: 0.10-0.96) and neurological issues (OR: 1.5.10-8; 95% CI: 1.5.10-8) in patients. If you're over the age of 60 (OR: 0.50; 95% CI: 0.27-0.92), have neurological issues (OR: 0.09; 95% CI: 0.04-0.18), or have emotional problems (OR: 0.06; 95% CI: 4.7.10-3-0.79), you're less likely to experience HTN urgency compared to the hypertensive emergency. Additionally, the risk of hypertensive urgency was solely elevated by headache (OR: 14.28; 95% CI: 3.32-61.47) [6].

The approach to diagnosing hypertensive emergencies relies on the evaluation of observed symptoms and signs. Useful diagnostic tests may include metabolic panels, urinalysis, B-natriuretic peptide levels, and cardiac enzymes. For those with suspected cardiac issues, an electrocardiogram (ECG) is advisable. A head computed tomography (CT) scan is recommended for acute neurological symptoms, while chest X-rays are beneficial for evaluating shortness of breath [7,8]. Treatment often involves IV vasoactive medications such as labetalol, esmolol, nicardipine, and nitroglycerin, which are known to be effective. If there is no sign of organ damage, it's preferable to lower BP gradually over a few days. Conversely, HTN during pregnancy requires prompt intervention. Pregnant women may be treated with nifedipine, methyldopa, or labetalol, and acute episodes might be managed with IV hydralazine or oral nifedipine [9,10].

Acute kidney injury, aortic coarctation, aortic dissection, chronic kidney disease, eclampsia, hypocalcemia, hyperthyroidism, pheochromocytoma, renal artery stenosis, and subarachnoid hemorrhage represent conditions that necessitate evaluation during hypertensive emergencies. Traditionally, such emergencies were linked to severe outcomes, including kidney damage, heart attacks, strokes, or even death. However, advancements in managing BP have led to a notable reduction in mortality rates over the past three decades. Despite these improvements, the long-term outlook for patients remains a concern, with a significant number encountering cardiac complications or strokes within a year following the emergency. The absence of prompt diagnosis and treatment of a hypertensive emergency can lead to severe consequences, such as kidney failure, loss of vision, heart attacks, or strokes [5,11].

For effective HTN management, a comprehensive medical history and physical examination are crucial. This process entails collecting detailed background information from various sources including family members, general practitioners, and previous medical records, as well as a thorough review of current medications. Symptom assessment is vital, focusing on issues such as headaches, shortness of breath, palpitations, and blurred vision. The physical examination should include BP measurement and evaluation of peripheral pulses. Retinal examinations play a pivotal role in diagnosing malignant hypertension (MHT), with patients exhibiting severe HTN requiring fundoscopic evaluations to detect retinal changes. For women of childbearing age, the possibility of pregnancy should be considered, necessitating a urine pregnancy test. Additionally, a bedside urine dipstick test for the presence of blood and protein is indispensable, particularly to identify nephropathy as a direct or indirect consequence of HTN. Treatment should commence immediately if a life-threatening condition is suspected [12]. The aim of this systematic review is to explore the management strategies for a hypertensive crisis, emphasizing the importance of a holistic approach to diagnosis and initial treatment planning.

## Review

Methods

This review was developed following the guidelines outlined in the Preferred Reporting Items for Systematic Reviews and Meta-Analyses (PRISMA) framework.

Search Strategy

**Figure 1:**
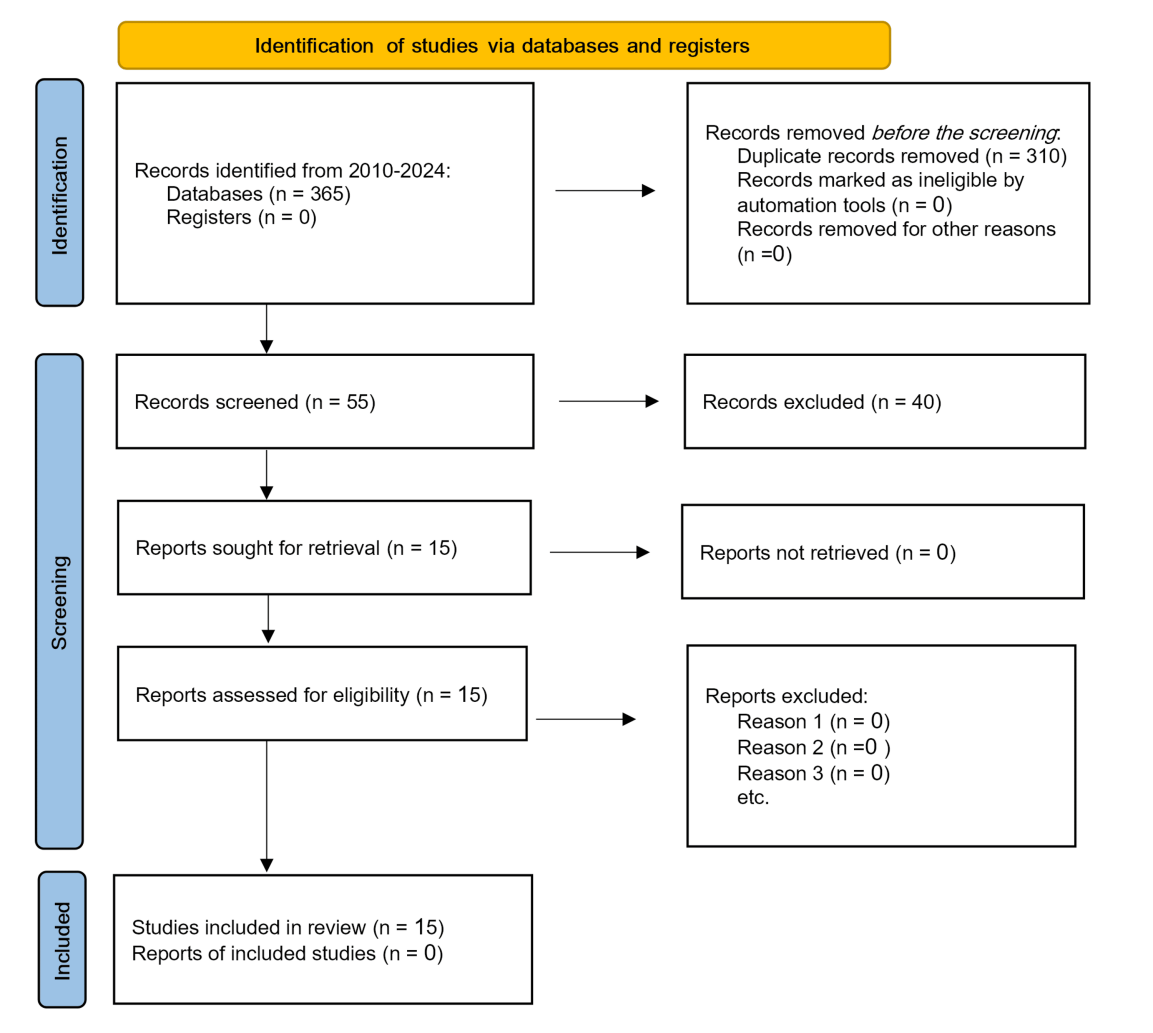
Study flow diagram following the PRISMA (Preferred Reporting Items for Systematic Reviews and Meta-Analyses) guidelines.

A comprehensive search was conducted in PubMed/Medline and Embase databases for the period from 2010 through January 2024, focusing on the management of hypertensive crises. The search utilized a combination of keywords, medical subject headings (MeSH), and heading phrases specific to the topic. The search was restricted to English-language publications. Further studies were identified by examining the references of the initially selected articles. Following the retrieval and review of full-text articles, 15 studies were deemed relevant and included in this review. The process of search screening and article selection adhered to the PRISMA guidelines (Figure 1).

Inclusion Criteria

The review focused on English-language articles and reviews that provided insights into the management of hypertensive crises. The included studies met the following criteria: (1) research articles or reports that diagnosed hypertensive crises, (2) studies demonstrating data integrity and the potential for reproducibility, and (3) articles focusing on management strategies for hypertensive crises.

Exclusion Criteria

Studies were excluded based on the following: (1) duplicate entries, incomplete or incorrect data, or data deemed unusable; (2) non-research materials such as comments, conference summaries, reports, and nonsystematic reviews; (3) outdated data or data covering the same region comprehensively; (4) studies with unclear sample sources; and (5) publications in languages other than English.

Study Selection Process

Our study selection process was meticulously organized to comprehensively identify and assess relevant literature. Initially, all discovered articles were imported into EndNote (Clarivate, London, UK) to facilitate the removal of duplicates. Following this step, the two reviewers accessed the EndNote library, now free of duplicates, to independently screen the articles by title and abstract according to pre-defined eligibility criteria. Subsequently, the studies preliminarily chosen by both reviewers were subjected to a full-text review. In instances of disagreement between the initial reviewers, a third reviewer was called upon to mediate and help achieve consensus. This approach ensured that each study deemed eligible underwent a thorough review of its full text by both initial reviewers independently. Whenever divergences in opinion arose, the third reviewer's input was sought to discuss and reconcile differences.

Ultimately, the complete texts of all studies that satisfied the inclusion criteria were compiled and retained for the final synthesis of the framework. This systematic and collaborative review process aimed to ensure the integrity and thoroughness of the study selection phase.

The search terms that were used in this study for identifying relevant literature on the management of hypertensive crises include "hypertensive crisis management", "acute severe hypertension treatment", "hypertensive emergencies strategies", "hypertension medical emergency", "blood pressure control in hypertensive crisis", "hypertensive crisis guidelines", "hypertensive emergencies medication", "management of hypertensive urgencies", "blood pressure targets in hypertensive crises", and "organ damage from hypertension". These terms were selected in order to address the condition comprehensively, from management strategies and treatment options to medication use as well as associated complications significantly contributing to a vast review of literature on this topic.

Primary outcome

The principal measure of interest was the effectiveness of various management strategies for hypertensive crises.

Data Extraction Process

In the initial phase of our research, we examined the titles and abstracts of 365 studies identified through our search strategy. This initial screening resulted in the selection of 55 papers for further consideration. Upon conducting a detailed review of the full texts of these papers, we narrowed our selection down to 15 papers that were deemed suitable for in-depth analysis. A summary of the data extracted from these papers is presented in Table 1.

**Table 1:** This summary includes essential information such as the authors and publication year, the identified causes of hypertension, and the management strategies discussed.

S. no.	Author and year	Causes of hypertension	Possible way of management
1	Papadopoulos et al., 2015 [13]	Patients experiencing hypertensive emergencies often face these due to specific triggers. These emergencies can be induced by the use of substances such as cocaine, amphetamines, phencyclidine, or monoamine oxidase inhibitors, which can lead to a pheochromocytoma-like or hyperadrenergic state. Additionally, suddenly stopping the use of clonidine or similar sympatholytic medications can also precipitate such crises.	Patients with acute aortic dissection necessitate rapid blood pressure management, typically through the administration of intravenous esmolol, aimed at reducing blood pressure within 5 to 10 minutes. The goal is to maintain the systolic blood pressure below 120 mmHg. Should the blood pressure not adequately decrease following beta-blocker treatment, vasodilators such as nitroglycerin or nitroprusside can be employed. For further blood pressure control, medications like clevidipine, nicardipine, or phentolamine may be administered intravenously. The initial dosage is often 5 mg, with subsequent doses administered at 10-minute intervals as required to achieve the desired blood pressure target.
2	Peacock et al., 2011 [14]	Hypertensive emergency accompanied by acute pulmonary edema.	In the management of hypertensive emergencies, medications such as intravenous nitroglycerin, clevidipine, or nitroprusside are commonly utilized. It is important to note that beta blockers are generally not advised for treating acute pulmonary edema. With the exception of acute aortic dissection cases, the protocol for hypertensive emergencies typically involves reducing the patient's blood pressure by 20% to 25% within the first minutes to an hour. Subsequently, the goal is to adjust blood pressure to 160/100 mmHg over the next two to six hours, followed by a cautious normalization within 24 to 48 hours. For intravenous nitroglycerin, the starting infusion rate is set at 5 micrograms per minute, with a maximum rate that can be adjusted up to 20 micrograms per minute. The initial rate for intravenous sodium nitroprusside begins at 0.3 to 0.5 micrograms per kilogram per minute, with a ceiling of 10 micrograms per kilogram per minute. Intravenous clevidipine starts at a rate of 1-2 milligrams per hour, escalating up to a maximum of 32 milligrams per hour if needed.
3	Rosendorff et al., 2015 [15]	Severe hypertension, with acute myocardial infarction or an unstable angina pectoris.	Patients with acute myocardial infarction or unstable angina pectoris should have a target blood pressure of less than 140/90 mmHg and a blood pressure of less than 130/80 mmHg upon hospital discharge. Avoid lowering diastolic blood pressure below 60 mmHg, as this may reduce coronary perfusion and exacerbate myocardial ischemia.
4	Varon et al., 2014 [16]	A hypertensive emergency results in acute renal failure.	The researchers compared the efficacy of intravenous fenoldopam and nicardipine in treating hypertension in 104 patients with renal dysfunction. The initial infusion rate for fenoldopam was 0.1-0.3 mcg/kg/min, while nicardipine was 5 mg/h with a maximum of 30 mg/h. In a 30-minute treatment, 92% of patients receiving nicardipine met their target systolic blood pressure.
5	Aronow, 2017 [17]	A hypertensive crisis with pre-eclampsia or eclampsia.	Patients with a history of hypertension are treated with medications such as hydralazine, labetalol, and nicardipine. However, angiotensin-converting enzyme inhibitors, angiotensin receptor blockers, direct renin inhibitors, and sodium nitroprusside are not advised. The initial dose is 20 mg of hydralazine, followed by 0.3 to 1.0 mg/kg of labetalol, for a total dose of 300 mg.
6	Espinosa et al., 2016 [18]	High blood pressure after surgery	The medication includes intravenous clevidipine, esmolol, nitroglycerin, and nicardipine.
7	Ayaz et al., 2016 [19]	A hypertensive emergency is characterized by a high plasma renin level.	Enalaprilat is administered intravenously. The first dose of enaliprilat is 1.25 mg given intravenously over five minutes. To achieve the desired blood pressure level, extra doses of intravenous enalaprilat of up to 5 mg every six hours may be given.
8	Potter and Schaefer, 2024 [20]	Hypertensive encephalopathy is related to high blood pressure.	The primary treatment for this condition is antihypertensive drug therapy to reduce mean arterial pressure (MAP) by 10% to 15% within the first hour, but no more than 25% of the original baseline. This cautious reduction lowers the risk of ischemic events and allows the brain's vasculature to heal. If the MAP falls below the hypertensive-adapted autoregulatory range, the risk of stroke and other ischemic complications increases. This conservative lowering does not apply to ischemic stroke, intracerebral hemorrhage, or aortic dissection.
9	Boulouis et al., 2017 [21]	Acute intracerebral hemorrhage (ICH) is a serious condition characterized by sudden and severe brain damage.	Unless contraindicated, administer IV labetalol (managed in a high dependency unit) and monitor with neuro-observations and renal function. IV nicardipine may be appropriate.
10	Sandset et al., 2021 [22]	Acute ischaemic stroke (AIS) is distinguished by a balance of decreased cerebral blood flow and increased cerebral edema, and routine antihypertensive therapy is typically unnecessary.	For acute therapy, administer IV labetalol, nicardipine, or glyceryl trinitrate (GTN). Choice of agent for long-term management in accordance with NG 136 (NICE guidelines for medication choices).
11	Maher et al., 2020 [23]	Considerations for BP-lowering therapy in subarachnoid hemorrhage (SAH).	Oral nimodipine may be used to treat SAH patients. Treatment for patients with concomitant hypertensive emergency state is determined by the associated diagnoses.
12	Zhou et al., 2023 [24]	Management of hypertension in acute aortic syndrome (especially type B).	In the acute phase, intravenous labetalol or esmolol is recommended. Once the heart rate is under control, IV nicardipine and/or nitroprusside can be given. If ß blockers are not tolerated, non-dihydropyridine CCB can be used to regulate heart rate. Oral medications were administered as tolerated. Long-term oral antihypertensive therapy helps maintain a target systolic BP of ≤120 mmHg.
13	Twiner et al., 2022 [25]	Considerations for BP-lowering therapy in acute coronary syndrome (ACS).	IV GTN and/or labetalol can be used. Nitroprusside should be avoided in ACS.
14	Van et al., 2019 [26]	Hypertension management in the presence of acute pulmonary edema.	IV GTN or nitroprusside combined with a loop diuretic, such as IV furosemide. Calcium channel blockers and intravenous labetalol are best avoided during the acute phase.
15	Buitenwerf et al., 2020 [27]	Management of hypertension caused by phaeochromocytoma/adrenergic crisis.	For α blockade, oral phenoxybenzamine (or doxazosin if unavailable) is recommended, followed by β blockade as needed. In the event of a crisis, IV phentolamine can be administered. Phenoxybenzamine is used to get ready for surgery. β blockers are used to manage persistent tachycardia. Benzodiazepines are prescribed to treat illicit drug-induced hypertension caused by cocaine or amphetamines. Fluid expansion and increased salt intake are recommended to avoid postural hypotension and tachycardia.

Discussions

High BP stands as the most prevalent and significantly alterable risk factor for cardiovascular disease and disability globally. There is strong evidence showing that antihypertensive medications effectively reduce the risk of cardiovascular diseases and damage to other organs. Meanwhile, instances of acute severe BP spikes have become less frequent than in past decades, likely due to improved screening, heightened awareness, and advanced management and treatment strategies for chronic HTN, particularly in developed nations. However, despite these advances, there are still cases of hypertensive crises, which pose serious life-threatening risks, potentially leading to swift organ damage or death [12]. This review focuses on the strategies for managing a hypertensive crisis, highlighting the critical need for timely and effective treatment in such emergencies.

A thorough medical history and physical examination are critical for the effective management of HTN, a prevalent health issue impacting around 50 million individuals in the United States and approximately one billion people globally. It is estimated that about 1% of these individuals will experience acute episodes of high BP during their lifetime. Severe HTN, also referred to as hypertensive crises, emergencies, and urgencies, is characterized by a sudden increase in BP leading to damage to vital organs. Immediate intervention to lower BP is necessary only for those patients who exhibit signs of acute damage to their organs [28].

Papadopoulos and colleagues (2015) identify several conditions as hypertensive emergencies, including dissecting aortic aneurysm, acute pulmonary edema, myocardial infarction, unstable angina, renal failure, intracranial hemorrhage, ischemic stroke, hypertensive encephalopathy, eclampsia, peri-operative HTN, pheochromocytoma crisis, and drug-induced hypertensive crises from substances like cocaine, amphetamines, phencyclidine, or monoamine oxidase inhibitors. Patients facing these emergencies need swift and effective treatment to reduce BP promptly, safeguard organ function, alleviate symptoms, minimize complications, and enhance overall outcomes. Without the use of antihypertensive drugs, the one-year mortality rate for individuals experiencing these emergencies can exceed 79%, with a median survival time of only 10.4 months. Treatment typically involves IV administration of medications such as clevidipine, nicardipine, or phentolamine, starting with an initial dose of 5 mg followed by additional doses of 5 mg every 10 minutes as necessary to maintain stable BP [13].

Espinosa and colleagues (2016) highlighted that in cases of hypertensive emergency accompanied by acute pulmonary edema, the drugs of choice include IV nitroglycerin, clevidipine, or nitroprusside. The treatment protocol specifically excludes the use of beta-blockers. The strategy recommends a rapid reduction of BP by 20% to 25% within the initial minutes, followed by a careful adjustment to reach a target of 160/100 mmHg, and ultimately, a return to normal BP levels over the span of 24 to 48 hours. The starting infusion rates are specified as 5 mcg/min for nitroglycerin, 0.3 to 0.5 mcg/kg/min for sodium nitroprusside, and 1-2 mg/hour for clevidipine. It is critical to note that beta-blockers are not recommended for use in this specific context [18].

Clevidipine (Cleviprex), a modern dihydropyridine calcium channel blocker formulated as a lipid emulsion for IV use, has received approval in the United States for lowering BP when oral administration is impractical or not preferred. This drug is notably effective in managing acute HTN both before and after cardiac surgery in adults. Its rapid onset and brief duration facilitate straightforward titration, enabling consistent BP management. Studies by Papadopoulos et al. (2015) and Espinosa et al. (2016) are in alignment with findings from Watson et al. (2018), which suggest oral nifedipine as a viable first-line therapy for pre-eclampsia, on par with IV hydralazine and labetalol. Clevidipine is also highlighted as a primary treatment choice for acute ischemic stroke and is considered for use in cases of intracranial hemorrhage. While managing hypertensive heart failure remains a challenge, clevidipine and enalaprilat are potential options for this demographic, although the evidence supporting their effectiveness is still emerging [4,29].

Esmolol hydrochloride, known for its IV administration and specificity as a cardioselective beta-1 adrenergic blocker, is employed across various clinical scenarios, including immediate care, alongside pre- and post-surgery settings [30]. Zhou et al. (2023) advocate for esmolol as the treatment of choice for acute aortic dissection, recommending a loading dose of 500 to 1,000 mcg/kg/min delivered over one minute, aiming to reduce systolic BP (SBP) to below 120 mmHg. In cases where BP persists at elevated levels, the administration of vasodilators, such as nitroglycerin or nitroprusside, is suggested. Additionally, esmolol is recommended for managing acute myocardial infarction, unstable angina pectoris, and significant HTN. For patients who are hemodynamically stable, the objective is to maintain BP below 140/90 mmHg, transitioning to a target of less than 130/80 mmHg by the time of hospital discharge. It is crucial to approach BP reduction cautiously to prevent reduced coronary perfusion, which could worsen myocardial ischemia [24].

Varon and colleagues (2014) examined the efficacy of clevidipine, fenoldopam, and nicardipine as preferred treatments for managing hypertensive emergencies and acute kidney injury. They noted that fenoldopam should be started at an infusion rate of 0.1-0.3 mcg/kg/min, while nicardipine begins at 5 mg/hour. In a study of 104 patients undergoing hypertensive emergency treatments with either nicardipine or labetalol, it was found that 92% of those treated with nicardipine achieved their target SBP within 30 minutes, a significant improvement over the 78% success rate observed with labetalol. Further analysis highlights fenoldopam's role as a rapid-acting, intravenously administered peripheral dopamine-1 receptor agonist that promotes vasodilation across coronary, renal, mesenteric, and peripheral arteries. This medication starts working within five minutes of administration, reaching its peak effectiveness at around 15 minutes. The suggested initial dosage of fenoldopam is 0.1 mcg/kg/min, with the option to adjust the dose in 15-minute intervals up to a maximum of 1.6 mcg/kg/min. Fenoldopam has been shown to enhance creatinine clearance, urine flow rates, and sodium excretion among hypertensive individuals with intact renal function, though its impact on reducing long-term morbidity or mortality remains unproven. While its side effects are generally mild, caution is advised when prescribing fenoldopam to patients with glaucoma [16,31-33].

Nicardipine, a second-generation calcium channel blocker, is distinguished by its high selectivity for vascular tissues and potent vasodilatory effects. It starts to work within 5 to 15 minutes of administration and has a duration of action lasting between 40 and 60 minutes. The recommended starting infusion rate is 5 mg per hour, which can be increased by 2.5 mg per hour every five minutes, up to a maximum rate of 15 mg per hour. In clinical trials, the most frequently reported side effects of nicardipine include thrombophlebitis, headaches, flushing, tachycardia, dizziness, and nausea [34,35].

Labetalol functions as both a nonselective beta-adrenergic and selective alpha1-adrenergic receptor blocker, making it an effective antihypertensive medication. The onset of its antihypertensive action occurs within two to five minutes following IV administration, with peak effects observed between 5 and 15 minutes, and the duration of efficacy extending from three to six hours. The administration protocol typically starts with a loading dose of 20 mg, with subsequent doses ranging from 20 to 80 mg administered at 10-minute intervals, until the target BP level is achieved. However, due to its potent nonselective beta-adrenergic blocking properties, labetalol is contraindicated in patients with asthma, uncontrolled heart failure, sinus bradycardia, or more than a first-degree heart block [36].

Hydralazine is an antihypertensive agent prescribed for managing primary HTN or severe HTN that demands urgent attention, including conditions like heart failure and pre-eclampsia or eclampsia. Aronow (2017) mentions that hydralazine, alongside labetalol and nicardipine, is recommended for treating hypertensive crises, including cases of eclampsia or pre-eclampsia. In contrast, certain medications such as angiotensin-converting enzyme (ACE) inhibitors, angiotensin receptor blockers, direct renin inhibitors, and sodium nitroprusside are advised against in these scenarios. The initial dosing of hydralazine can go up to 20 mg, with subsequent doses ranging from 0.3 to 1.0 mg/kg, not to exceed a total dose of 300 mg [17,37].

Enalaprilat injection is indicated for the management of HTN when oral therapy is not feasible. It represents the active form of enalapril maleate and is designed for IV use. By competing with ACE, enalaprilat prevents the conversion of angiotensin I to angiotensin II, a mechanism that is central to its antihypertensive effect. This inhibition encompasses the full scope of enalaprilat's use, including its mechanism of action, potential side effects, pharmacological properties, dosage guidelines, contraindications, safety warnings, necessary precautions, monitoring requirements, potential toxicity, and drug interactions, all of which are essential for comprehensive care by an interprofessional team. Ayaz et al. (2016) noted that for patients exhibiting high plasma renin activity, enalaprilat is initially dosed at 1.25 mg and administered over five minutes. Subsequent doses can be given every six hours as needed to achieve and maintain the desired BP control [19,38].

For the initial management of hypertensive emergencies, parenteral antihypertensive medications are recommended. Oral antihypertensive agents are generally not favored at this stage due to their inability to be precisely titrated and their potential for delayed action. Preferred IV antihypertensive drugs include nicardipine, starting at a rate of 5 mg/hour and potentially increasing to a maximum of 15 mg/hour, labetalol, fenoldopam, and clevidipine. These medications are crucial for quickly reducing BP to safe levels. Oral antihypertensives may be introduced once the initial IV therapy has been gradually reduced and stopped, typically within 8 to 24 hours of achieving the desired BP goals [39].

Observational research has established a connection between elevated BP in the acute phase of intracerebral hemorrhage and an increased likelihood of hematoma expansion, peri-hemorrhagic edema, and a higher risk of neurological decline, disability, or mortality in more than two-thirds of affected individuals. Lattanzi et al. (2017) and Kapinos and Hanley (2018) have contributed valuable perspectives on managing high BP after the onset of spontaneous ischemic heart failure (ICH), a topic that has sparked debate in the context of acute stroke care. Their findings indicate that promptly initiating intensive BP reduction to below 140 mm Hg is not only safe but also helps in reducing heart failure without adversely impacting neurological outcomes. Despite these benefits, such an approach did not lead to a significant decrease in the rate of death or disability at three months among patients with elevated BP, compared to a more conservative treatment strategy. Present-day guidelines advocate for the acute reduction of SBP to 140 mm Hg in patients with an SBP range of 150 to 220 mm Hg. For those with an SBP exceeding 220 mmHg, a more aggressive reduction is recommended, involving continuous IV infusion and close monitoring. The integration of auto-regulatory indices and neuroimaging markers of ischemic risk into treatment planning can enhance BP management by identifying safe thresholds for BP reductions that do not compromise brain metabolism [40-43].

Nitroglycerin, a rapid-onset vasodilator, is widely used in emergency departments for treating angina, chest pain associated with acute coronary syndromes, and other conditions including acute heart failure, pulmonary edema, and aortic dissection. It metabolizes into nitric oxide, a potent vasodilator that induces venodilation at lower doses and arteriodilation at higher doses. While nitroglycerin was traditionally administered in the form of a sublingual tablet or spray, its IV application in emergency settings has become more common due to its predictable pharmacokinetics [44].

The review aimed to evaluate the quality of research in a specific field, focusing on English-language studies, which may have introduced linguistic bias and overlooked significant non-English research due to publication bias. The exclusion of studies not in peer-reviewed journals, along with case reports and non-randomized trials that lacked methodological rigor, further limited the review's comprehensiveness. Standardized tools like the Newcastle-Ottawa Scale and the Cochrane Risk of Bias tool were used to assess study quality, revealing various biases including conflicts of interest, inconsistent reporting, short follow-up periods, and small sample sizes. The review also faced challenges in synthesizing data due to heterogeneous study designs, demographics, interventions, and outcomes, compounded by potential biases from non-blinded studies and inadequate control for confounders. Consequently, the review's reliability was compromised, failing to cover all relevant topics adequately due to these multiple constraints.

The results stress the importance of tailoring HTN care to each patient by taking their unique set of medical history, current symptoms, and other variables into account, such as the intensity of their hypertensive crisis and how well they react to medicine. Efficacy, safety, start of action, and patient tolerance should be considered by clinicians when selecting antihypertensive medications. HTN crisis care standardization includes creating and revising clinical guidelines, improving healthcare provider education and training, standardizing monitoring protocols, promoting a multidisciplinary approach, and educating patients on the importance of taking their medications as prescribed, making lifestyle changes, and getting regular BP checks.

## Conclusions

Clinical practice in the management of hypertensive crises is outlined throughout the book with specific recommendations. Prioritizing symptoms and possible end-organ damage stresses the significance of quick evaluation and triage. Beta-blockers, beta-adrenergic blockers, nitroglycerin, esmolol, and sodium nitroprusside are the first-line agents. Important follow-up and monitoring include taking a BP reading every 30-60 minutes after stabilization and every 5-15 minutes during the first treatment. After end-organ damage has been monitored on a regular basis, the next step is to switch to oral antihypertensive medications once BP is under control.

In order to control BP in the long run, it is advised that patients get information and make adjustments to their lifestyle, such as eating healthier and exercising more frequently. Healthcare providers undergo ongoing education on the most recent standards and norms, and standardized practices are formally established.

Research priorities for the future include determining which treatments are safest and most effective, finding predictive biomarkers, creating early detection tools, studying the effects of lifestyle modifications, and evaluating patient education initiatives. It also delves into the management of certain demographics, like pregnant women and the elderly. Research on HTN crisis prediction and personalized treatment strategies is underway, with a focus on technology and telemedicine for early detection and management. Healthcare practitioners can better manage hypertensive crises, improve patient outcomes, and fill in knowledge gaps by concentrating on these areas of suggestion and study.
